# The Role of Electroencephalography in the Prognostication of Clinical Outcomes in Critically Ill Children: A Review

**DOI:** 10.3390/children9091368

**Published:** 2022-09-08

**Authors:** Carley A. Gilman, Courtney J. Wusthoff, Réjean M. Guerriero

**Affiliations:** 1 Division of Pediatric Neurology, Department of Neurology, Washington University in St. Louis, St. Louis, MO 63110, USA; 2 St. Louis Children’s Hospital, St. Louis, MO 63110, USA; 3 Division of Child Neurology, Department of Neurology, Stanford University, Palo Alto, CA 94305, USA; 4 Lucile Packard Children’s Hospital Stanford, Palo Alto, CA 94304, USA

**Keywords:** neurocritical care, EEG, outcomes, pediatric critical illness, seizures, status epilepticus

## Abstract

Electroencephalography (EEG) is a neurologic monitoring modality that allows for the identification of seizures and the understanding of cerebral function. Not only can EEG data provide real-time information about a patient’s clinical status, but providers are increasingly using these results to understand short and long-term prognosis in critical illnesses. Adult studies have explored these associations for many years, and now the focus has turned to applying these concepts to the pediatric literature. The aim of this review is to characterize how EEG can be utilized clinically in pediatric intensive care settings and to highlight the current data available to understand EEG features in association with functional outcomes in children after critical illness. In the evaluation of seizures and seizure burden in children, there is abundant data to suggest that the presence of status epilepticus during illness is associated with poorer outcomes and a higher risk of mortality. There is also emerging evidence indicating that poorly organized EEG backgrounds, lack of normal sleep features and lack of electrographic reactivity to clinical exams portend worse outcomes in this population. Prognostication in pediatric critical illness must be informed by the comprehensive evaluation of a patient’s clinical status but the utilization of EEG may help contribute to this assessment in a meaningful way.

## 1. Introduction

Electroencephalography (EEG) is increasingly utilized in pediatric critical care [1,2]. This modality is primarily used to detect seizures in at-risk pediatric patients. However, there is additional utility in evaluating other characteristics of an EEG tracing to understand the cerebral function and gain insight into prognosis [3]. Recently, research has evaluated EEG data more broadly to better characterize cerebral function in critical illness. Through this work, a spectrum of function–dysfunction has emerged even within the same disease process. The clinical significance of EEG findings is being studied in the context of both acute and long-term functional outcomes in order to provide improved clinical guidance for providers and families. This review examines the current literature on EEG utilization in critically ill children, with particular attention paid to patient outcomes (for a summary, see Table 1). Areas of further research are suggested to better understand these relationships.

## 2. Seizure Presence, Duration and Burden

### 2.1. Seizures

Seizures are a common complication of critical illness. The incidence of any seizure, including both clinical and nonconvulsive seizures, in critically ill children, has been reported in 16–46% of patients (mean 32%, median 36%) [4,5,6,7,8,23,24,25,26,27,28]. Furthermore, of patients with witnessed clinical seizures at hospital presentation, 22–33% will have seizure recurrence during their hospitalization [27,29,30]. In addition to clinical seizures, nonconvulsive seizures are also of concern in this population. Children with acute mental status changes during critical illness are often evaluated with EEG to understand if nonconvulsive seizures are contributing to depressed mental status. The incidence of nonconvulsive seizures in all children on continuous EEG monitoring is reported to be between 10% and 42% (mean 23%, median 16.3%) [5,6,7,8,9,24,26]. Given the high incidence of nonconvulsive seizures among critically ill children with acute mental status changes, EEG monitoring is recommended to quickly identify and treat seizures in this vulnerable group.

A young age of the patient, particularly under the age of 2 years, is a significant risk factor for acute seizures during illness [5,23,28,31,32,33]. Abend and colleagues found that 56% of patients younger than 1 year who underwent clinically indicated EEG monitoring had seizures during their EEG recording [23]. Additionally, head trauma, particularly from a non-accidental mechanism or resulting in severe injury on neuroimaging, has been independently shown to be a risk factor for seizures [5,10,26,28,31,33,34,35]. The identification of these risk factors allows providers to increase vigilance and surveillance of seizures in these populations. Where EEG monitoring is available on a limited basis, these factors can identify children at the highest risk and, therefore, the highest priority for EEG. While seizures are common in critical illness, not all children with the same underlying illness or demographics experience this complication. Therefore, studying the patients who experience seizures as a subgroup is imperative, both to understand the association of seizures with acute outcomes and as possible predictors of long-term functional outcomes.

It remains controversial to what degree seizures independently impact outcomes by increasing metabolic demand in vulnerable parenchyma or whether seizures merely reflect a worse underlying injury. The best available studies on humans rely on observational data, which has inherent limitations. At the same time, accumulating evidence suggests a dose-dependent, or possibly a threshold relationship, between seizures and worse outcomes.

Self-limited, isolated seizures have a very limited impact on outcomes. In the short-term, patients who experience seizures during their hospital course do not have increased in-hospital mortality compared to those without seizures [6,7,9,11,12,13,14,24]. The assessment of long-term functional outcomes most often relies on The Glasgow Outcome Scale-Extended (GOS-E) and Pediatric Cerebral Performance Category (PCPC) scales. The Adaptive Behavior Assessment System II (ABAS-II) to evaluate the long-term adaptive function and the Kings Outcome Scale in Childhood Head Injury (KOSCHI) to understand broader neurologic functioning specifically in the brain injury population are also reported. Robust data have emerged to suggest that functional outcomes using the KOSCHI, GOS-E and PCPC scores do not differ between those who experience isolated seizures and those who do not [5,9,11,14].

Seizures considered on a binary basis, simply as present or absent, are not associated with an increased risk of adverse outcomes. However, increasing seizure burden, or the cumulative duration of seizures during an acute illness, has been associated with increased functional deficits at discharge, including gross motor and mental status impairments [8,25]. Seizure burden and duration may be a better prognostic variable than seizure presence during critical illness. The most extreme example of this may be the impact of status epilepticus.

### 2.2. Status Epilepticus

Status epilepticus (SE) reflects prolonged seizure duration. While historically defined as a seizure lasting greater than 30 min or greater than 30 min of seizure time within an hour period, more recent definitions have proposed a minimal duration of 10 min of continuous seizure activity or 12 min of cumulative seizures in any one-hour period [36]. Nonconvulsive SE is a particular concern in the critical care setting, given limited clinical exams in intubated and sedated children who are at risk for cerebral dysfunction. The incidence of SE in all critically ill children monitored on EEG has been reported to be between 6% and 35% of patients (mean 18.7%, median 18.4%), although sample sizes varied [5,6,8,12,13,15,37,38,39]. In some cases, SE may lack clinical signs due to the use of neuromuscular blockade. However, Topjian and colleagues found that only 17% of patients with electrographic-only seizures in their cohort were simultaneously receiving a pharmacologic paralytic [9]. This suggests that the lack of motor features in these seizures may not solely be due to iatrogenic factors. Nonconvulsive seizures should be considered in all patients with depressed mental status of uncertain etiology, not just those receiving paralytics.

SE has been associated with increased in-hospital mortality independently of the underlying disease process in children with acute encephalopathy (OR 2.42–7.76) [9,12,13]. This, however, has been variably replicated as several studies have shown no association between SE and mortality in children with critical illness of any etiology [6,8]. Given these conflicting reports, it is currently difficult to make clinical decisions using the published data. Much like seizure burden, the duration of status epilepticus may be accounting for the discrepancies in the literature surrounding mortality. This has yet to be evaluated and should be investigated within specific etiologies to better understand if there is a correlation between status and outcomes.

In addition to an association with short term-mortality, the long-term association of SE with functional outcomes in children has been evaluated. Patients with SE during illness have worse adaptive behavior on the ABAS-II at discharge than patients with only short seizures [4]. Several groups have found that the presence of SE in critically ill children was associated with worse functional outcomes based on GOS-E and PCPC scores at discharge (OR 2.17–17.3) [6,8,9,11]. This indicates significant gross motor and/or mental status impairments in children with acute seizures compared to their pre-hospital conditions alone. In children with traumatic brain injury, the presence of status epilepticus was independently associated with more significant global disability as measured by the KOSCHI score at hospital discharge [5]. Finally, children who experience SE as part of their critical illness have a lower quality of life scores on the Pediatric Quality of Life Inventory (PedsQL) [11]. This finding likely reflects the experience of new neurologic deficits in patients and their families.

While the effects of status epilepticus on short-term mortality remain unclear, the impact on broader functional outcomes at discharge has a clearer negative association. Even without definitive proof of a causal relationship, it is reasonable in light of this evidence to treat seizures early and consistently in children who experience a high seizure burden/SE during critical illness to potentially improve their functional outcomes.

### 2.3. Special Population: Children on ECMO

**Figure 1:**
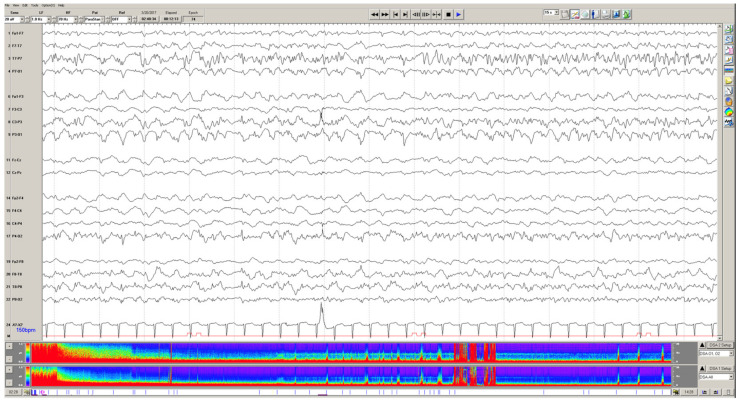
Status epilepticus in a 7-year-old male with no neurologic premorbidity, cannulated to VA ECMO for respiratory failure and shock. The EEG above demonstrates the left posterior quadrant focal status epilepticus. The density spectral array (color panel at the bottom) illustrates subsequent seizures during this 12-h recording epoch as denoted by red peaks throughout the array.

There are several specific pediatric populations that have a higher risk of seizures secondary to the underlying etiology of their critical illness. Children on extracorporeal membrane oxygenation (ECMO) are at particularly high risk for acute symptomatic seizures. This is related to both the underlying disease that necessitates the child needing ECMO (e.g., respiratory or cardiac failure), as well as the inherent risk of both ischemic and hemorrhagic strokes while using this therapeutic modality. Figure 1 illustrates a patient example of persistent seizures in a child on ECMO with a normal head CT and no premorbid neurologic condition, underscoring the importance of standardized monitoring in these children.

In children on ECMO, clinical features of seizures are particularly difficult to detect due to the higher utilization of neuromuscular blockade, confounding this clinical evaluation [40]. Thus, many of these patients are placed on continuous EEG monitoring, particularly during their immediate post-cannulation period. The incidence of seizures in these patients on EEG has been found to range from 18% to 23% [7,32,40,41]. The veno-arterial (VA) modality has a more significant risk for seizures than veno-venous (VV) ECMO, given the higher risk of thromboembolism, creating a structural epileptic focus [40].

Children who experience acute seizures during ECMO utilization were also more likely to have subsequent abnormal brain imaging [8,32,42]. These imaging abnormalities often indicate a structural etiology for seizure generation. The association of in-hospital mortality in children on ECMO who experience seizures versus those who do has not been clarified. The Pianto study in 2013 did not show an association between seizures and mortality [42], but two other studies did observe this link [32,40]. The Lin group also found that children on ECMO who experience acute seizures were more likely to have worse PCPC outcomes at discharge [40]. While the current evidence regarding outcomes of children on ECMO with seizures is limited, the results above do suggest a trend towards a higher risk of poor outcomes in this group. However, this association could be secondary to an underlying structural injury, including acute ischemic and/or hemorrhagic stroke while on ECMO, which causes both seizures and long-term deficits. The independent association of seizures on outcomes in this population remains undetermined.

## 3. Background Features

### 3.1. Organization and Sleep Architecture

The background characteristics of an EEG recording can indicate the overall health and function of a patient’s brain parenchyma. Given the robust data that can be gathered using EEG, clinical prognostication and research efforts have turned to the evaluation of EEG background to better understand cerebral function in correlation with overall clinical status in comatose children. For both research and clinical characterization purposes, EEG background features can be divided into four main categories: (1) normal, (2) slow/disorganized, (3) discontinuous or burst suppressed and (4) featureless. Most children (51–84%) with acute encephalopathy of any etiology have slow or disorganized backgrounds upon EEG initiation [6,15,27,43], although some have normal EEG backgrounds [27].

A normal EEG background has been shown to be a reassuring feature against the development of acute seizures [27,40]. Conversely, an abnormal EEG background was a risk factor for seizures in post-cardiac arrest patients, children on ECMO and in broader pediatric critical illness [30,40,44]. Topjian and Fung’s group found that patients with discontinuous/burst suppression or attenuated/featureless EEG backgrounds had a higher likelihood of in-hospital mortality (OR 8.39–41) [6,9]. When evaluating the same population for longer-term outcomes, the children with worse EEG backgrounds were also more likely to have unfavorable GOS-E and poorer PCPC outcomes at discharge (OR 4.74–28) [6,9]. As such, EEG background organization can be used, albeit cautiously, to understand patients who have a greater risk of long-term impairments. Combining EEG with imaging may further improve the prognostic yield, as demonstrated by Smith et al. following pediatric cardiac arrest. Combining a severely abnormal EEG with diffusion restriction on MRI was associated with a poor outcome (defined as a PCPC of 4–6) and increased the area under the receiver operator curve from 0.86 to 0.94 with EEG alone [45].

Sleep architecture, particularly the presence of sleep spindles, is another background EEG feature that suggests reassuring cerebral function. In children with traumatic brain injury, Vaewpanich and colleagues found that the lack of normal sleep architecture was associated with poorer outcomes on the GOS-E and Speech Pathology Neurocognitive/Functional Evaluation (SPFNE) at discharge and outpatient follow-up [10].

Overall, background organization and sleep features can give insight into cerebral health across acute illness etiologies and can be used for acute clinical evaluation as well as a prognostic tool for families and providers. This makes a comprehensive evaluation of EEG data, in addition to paroxysmal features, such as seizures, valuable in the clinical setting.

### 3.2. Special Population: Children with Cardiac Arrest

The pediatric cardiac arrest population has been a specific focus of EEG background evaluation. In these children, studies have reported a normal background in 3–10%, slow disorganized in 45–49%, burst suppression in 19–25% and attenuated/featureless in 16–33% of patients [14,16,17]. Research groups found that the latter two EEG background categories were associated with worse functional outcomes on the PCPC and a higher risk of in-hospital death [14,16,17,18]. Normal or continuous but slow EEG backgrounds after cardiac arrest have been associated with favorable PCPC outcomes in children [18,19].

The presence or absence of sleep spindles following pediatric cardiac arrest may also help guide prognostication. Ducharme-Crevier identified sleep spindles in 29% of patients, with an average time to spindle appearance of approximately 12.2 h after achieving the return of spontaneous circulation [20]. The presence of sleep spindles and normal sleep patterns in this population has been correlated with better functional outcomes and little to no change in premorbid PCPC scores at discharge [19,20]. Further, recent data reported a lack of sleep spindles was associated with short-term mortality (OR 7.61) and worse functional outcomes based on the PCPC score, GOS-E scale and the Speech Pathology Neurocognitive/Functional Evaluation (SPFNE) (OR 6.46) at discharge [14,17].

## 4. Periodic Features

### 4.1. Epileptiform Discharges

Paroxysmal features on EEG, such as periodic patterns and epileptiform discharges, are common in continuous EEG studies in a pediatric ICU setting, but there is limited literature on the relationship of these patterns with the outcome. Fung and colleagues reported epileptiform discharges were present in up to 24% of critically ill children during the first 30 min of EEG recordings [6]. The presence of these abnormalities was associated with an increased risk of acute seizures [42]. However, Hosain et al. found the prevalence of epileptiform discharges to be 12% in patients that did not have electrographic seizures on EEG recording [39]. While this was confounded by the short length of the recording in this study, it does suggest a higher tendency for these epileptiform abnormalities in critically ill children regardless of the presence of seizures.

In terms of the impact of these features on clinical outcomes, Fung et al. found that interictal epileptiform discharges and ictal-interictal epileptiform patterns were independently associated with unfavorable functional outcomes on GOS-E and PCPC [6]. This study also found an association between these EEG findings and a higher risk of in-hospital mortality [6]. As this dataset was limited by small sample size, larger datasets are needed to replicate these findings and establish a clear association between epileptiform discharges and outcomes.

### 4.2. Generalized Periodic Discharges

**Figure 2:**
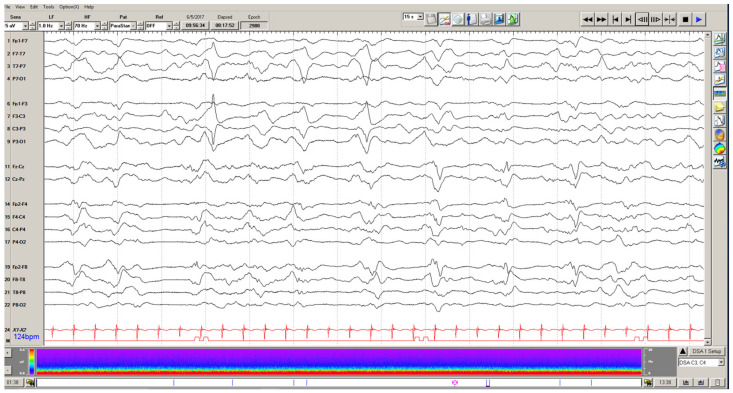
Generalized periodic discharges in a 7-year-old, previously healthy female with a grazing gunshot wound to the left posterior aspect of her head. EEG demonstrates evolution from a burst suppression pattern to an invariant pattern of periodic discharges happening every 2 s.

Generalized periodic discharges (GPDs), commonly seen in cerebral dysfunction of various etiologies, may also have implications for outcomes. GPDs are referred to as being on the ictal-interictal continuum. Figure 2 demonstrates the appearance of these discharges on EEG. The early literature on adult patients with intracranial hemorrhage suggested that GPDs were associated with worse modified Ranken Scale (mRS) scores at discharge [46,47]. However, in a recent matched-case control study, GPDs in hospitalized adults with EEG data were associated with nonconvulsive seizures and nonconvulsive status epilepticus but not with worse GOS outcomes or higher risk of mortality on multivariate analysis [48]. Jadelja and colleagues investigated this link further and found an increased risk of in-hospital mortality in adults with GPDs in the setting of worse mental status [49]. Abnormal mental status in the setting of GPDs suggests that the discharges are more ictal than interictal on the continuum, thus reinforcing the link between prolonged ictal states (i.e., seizure) and poor outcomes [36,50].

In the pediatric setting, Akman and colleagues demonstrated GPDs in about 7% of critically ill pediatric patients, excluding those with pre-existing epilepsy [51]. Forty-seven percent of those patients had subclinical seizures within 48 h prior to the presence of GPDs. Additionally, clinical seizures occurred in 66% of patients after the occurrence of GPDs, suggesting a strong link between the presence of GPDs and an increased risk of acute seizures, as seen in the adult literature [51]. Therefore, the association of GPDs with increased risk of seizure has become clearer across the literature, and acute surveillance for seizures in these patients is warranted.

In evaluating the outcomes of acutely ill children with GPDs, 23% died in the hospital, but 33% returned to baseline mRS score or had only mild disability at discharge [51]. The generalization of these findings is limited by a small dataset. Therefore, the independent impact of these findings on pediatric outcome data remains uncertain, likely confounded by where they sit on the ictal–interictal continuum.

### 4.3. Macroperiodic Oscillations

There is growing interest in using quantitative methods to dive deeper into EEG data, including understanding the computational makeup of epileptiform periodic patterns [52]. Additionally, spreading cortical depolarizations, occurring every 10 to 100 s, measured with intracranial electrocorticography (EEG on the brain surface), were independently associated with poor neurologic outcomes in adults with traumatic brain injury [53]. In pediatric patients, a slower periodic and oscillatory pattern has been linked to outcomes. Macroperiodic oscillations (MOs) oscillate over several minutes, occur preferentially in younger children with acquired brain injuries and may be associated with seizures [54]. The strength of the MOs signal has recently been linked to a poor outcome based on PCPC at hospital discharge [55]. Given the relatively recent identification of MOs, further research is needed to substantiate and understand the independent link between these slow EEG patterns and long-term clinical outcomes.

## 5. Electrographic Reactivity

**Figure 3:**
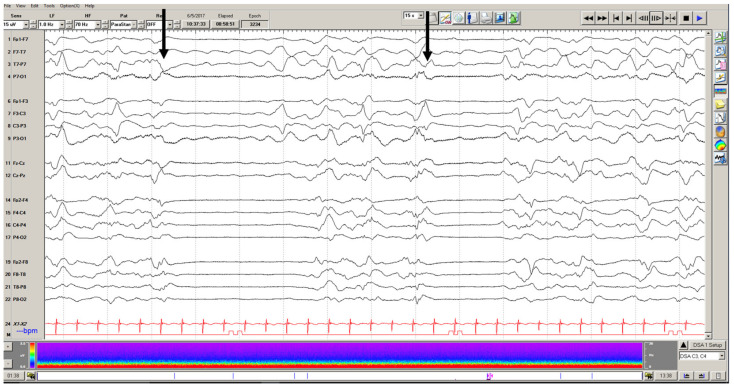
Reactivity testing in the same 7-year-old girl from Figure 2. When noxious stimulation was administered (black arrows), there was a reliable electro-decrement of the EEG recording. This was interpreted as an arousal response despite no clinical reactivity on exam. The patient was found to have a large left hemispheric intraparenchymal hemorrhage that was evacuated. The patient recovered and was transferred to neurorehabilitation with hemiparesis.

Electrographic reactivity is defined as a change in voltage or frequency of the EEG background in direct response to a stimulus, which can be observed despite sedation (Figure 3). When evaluating a comatose patient, reactivity is a feature that is assessed during an EEG recording to understand cerebral function and response to the external environment. This feature has been studied as another potential predictor of short and long-term outcomes in critical illness. The most robust research in this area has been conducted on adults. In a 2018 systematic review of adults with impaired consciousness for any reason, lack of reactivity on EEG was associated with poorer outcomes at discharge as measured by the GOS, mRS and CPC (Cerebral Performance Category) scores [56].

Given these findings in adults, several studies have looked for similar findings in pediatric patients. The most robust pediatric data has emerged from the pediatric cardiac arrest population. Reactivity on EEG in this group has been reported to be between 36% and 46% [16,17]. Lack of reactivity has been consistently associated with worse PCPC scores at discharge compared to their pre-hospital functioning and mortality, whereas patients that demonstrate reactivity on EEG had more favorable outcomes (stable or minimally changed PCPC scores) [14,16,17,19,21]. Prajongkit et al. evaluated children after cardiac arrest who were undergoing therapeutic hypothermia. While their pilot study was limited by number, they similarly noted that all patients with EEG reactivity undergoing this therapeutic modality had good functional outcomes on their CPC scores at discharge [21]. PCPC scores were derived from CPC scores for the pediatric population and are functionally similar.

More broadly, in comatose children of any etiology, the incidence of reactivity on EEG has been reported to be between 42% and 69% [6,22]. EEG reactivity in these comatose children of heterogeneous etiologies was associated with better functional outcomes on post-hospitalization PCPC scores [52]. Conversely, a lack of reactivity portended worse functioning (on both PCPC and GOS-E scales), as well as new neurologic deficits at discharge (OR 6.70) and mortality (OR 7.40) [6,8,22]. This relationship was also identified in children older than 1 month of age with hypoxic-ischemic encephalopathy from various etiologies [57]. Based on these studies, the presence of reactivity patterns of critically ill children on EEG can provide both short and long-term prognostication, regardless of the underlying disease process.

## 6. Conclusions

Critically ill children are at high risk for electrographic seizures given their often diffuse cerebral dysfunction in addition to any acquired structural injuries. EEG has been used to assess seizures in these patients, which has led to improved identification and treatment. Increased EEG utilization has highlighted certain EEG background features, such as background organization and sleep features, that add additional value in prognosticating short and long-term outcomes. These studies have demonstrated clear correlations with poorer functional outcomes such as moderate–severe EEG background disorganization, interictal discharges, lack of EEG reactivity and lack of normal sleep architecture. As such, EEG can be another tool for clinicians to gather data about a patient’s clinical status and help guide prognostication.

There remain several other EEG characteristics that require further exploration with a lens focused on acute and long-term outcomes. Future studies should include detailed investigations into the neurophysiologic impact of seizure burden on outcomes, specific structural drivers of outcomes in high-risk populations (e.g., children on ECMO) and more robust studies to replicate data pertaining to epileptiform discharges and the effects on long-term function. This work is critical to understanding how we can use comprehensive EEG data clinically and how this information can aid practitioners in clinical decision-making and counseling.

## Figures and Tables

**Table 1:** EEG features and the association with clinical outcomes [4,5,6,7,8,9,10,11,12,13,14,15,16,17,18,19,20,21,22].

	Seizures	Status Epilepticus	Background		Sleep Spindles		Reactivity		Epileptiform Discharges	Generalized Periodic Discharges
			Normal	Abnormal	Present	Absent	Present	Absent		
Decreased mortality/ Improved function			Nishisaki et al., 2007 Ostendorf et al., 2016		Ostendorf et al., 2016 Ducharme-Crevier et al., 2017		RamachandranNair et al., 2005 Ostendorf et al., 2016 Prajongkit et al., 2019			
No association with mortality/ function	Jette et al., 2006 Abend et al., 2013 Arndt et al., 2013 Wagenman et al., 2014 Sanchez Fernandez et al., 2017 Fung et al., 2019 Fung et al., 2021 MacDarby et al., 2021	Gwer et al., 2012 Payne et al., 2014 Fung et al., 2021								Akman et al., 2013
Increased mortality/ Worse function		Abend et al., 2013 Arndt et al., 2013 Topjian et al., 2013 Payne et al., 2014 Wagenman et al., 2014 Abend et al., 2015 Sanchez Fernandez et al., 2017 Fung et al., 2021		Nishisaki et al., 2007 Topjian et al., 2013 Ducharme-Crevier et al., 2017 Fung et al., 2019 Fung et al., 2021 Topjian et al., 2021		Vaewpanich et al., 2016 Topjian et al., 2021		Mandel et al., 2002 RamachandranNair et al., 2005 Topjian et al., 2016 Fung et al., 2019 Fung et al., 2021 Topjian et al., 2021	Fung et al., 2019 Fung et al., 2021	

## Data Availability

No new data were created or analyzed in this study. Data sharing is not applicable to this article.
